# Sleep-Wake Cycle in Young and Older Persons with a Lifetime History of Mood Disorders

**DOI:** 10.1371/journal.pone.0087763

**Published:** 2014-02-25

**Authors:** Rébecca Robillard, Sharon L. Naismith, Kristie Leigh Smith, Naomi L. Rogers, Django White, Zoe Terpening, Tony K. C. Ip, Daniel F. Hermens, Bradley Whitwell, Elizabeth M. Scott, Ian B. Hickie

**Affiliations:** 1 Clinical Research Unit, Brain & Mind Research Institute, The University of Sydney, Camperdown, New South Wales, Australia; 2 Healthy Brain Ageing Clinic, Brain & Mind Research Institute, The University of Sydney, Camperdown, New South Wales, Australia; 3 Concord Clinical School, The University of Sydney, Concord, New South Wales, Australia; Hôpital du Sacré-Coeur de Montréal, Canada

## Abstract

Considering the marked changes in sleep and circadian rhythms across the lifespan, age may contribute to the heterogeneity in sleep-wake profiles linked to mood disorders. This study aimed to investigate the contributions of age and depression severity to sleep-wake disturbances. The Hamilton Depression Rating Scale (HDRS) was administered to assess current symptoms severity in 238 persons with a history of a mood disorder between 12 and 90 years of age (y.o.). Actigraphy was recorded over five to 22 days. Regression analyses and analyses of variance [age (12–19 y.o., 20–39 y.o., 40–59 y.o., and ≥60 y.o.) by depression severity (HDRS< and ≥8)] were conducted. The 12–19 y.o. and 20–39 y.o. groups had a delayed sleep schedule and acrophase compared to all other groups. The ≥60 y.o. group had a lower rhythmicity and amplitude (p≤.006) than the 12–19 y.o. group (p≤.046). Participants with a HDRS≥8 spent longer time in bed, had later sleep offset times and had lower circadian rhythmicity than those with a HDRS<8 (p≤.036). Younger age and higher HDRS score correlated with later sleep onset and offset times, longer time in bed, higher WASO, lower sleep efficiency and later acrophase (p≤.023). Age was a significant predictor of delayed sleep and activity schedules (p≤.001). The profile of sleep-wake cycle disturbances associated with mood disorders changes with age, with prominent sleep phase delay during youth and reduced circadian strength in older persons. Conversely, disruptions in sleep consolidation seem more stable across age.

## Introduction

An increasing body of evidence suggests that the symptomatology and pathophysiological profile of depression during youth differs to that seen during old age, notably in terms of genetic and vascular factors, as well as cognitive impairment [1]–[4]. Despite the potential role of sleep-wake disturbances in the pathogenesis and maintenance of mood disorders and the extensive changes in the sleep-wake cycle across the lifespan, little attention has yet been given to the potential interactions between depressive illness and sleep-wake cycle modifications across various ages.

Decreased energy levels, apathy and daytime fatigue are hallmark features of depressive syndromes [5] and are likely to result from, and be further exacerbated by, sleep-wake disturbances. The low levels of physical activity commonly associated with depression [6]–[9] comes as a potential modulating factor of this bidirectional relationship between daytime and nighttime depressive symptoms. During the day, low energy levels, fatigue and apathy may decrease the motivation to engage in an active lifestyle. This may, in turn, perpetuate sleep-wake problems and fatigue by negating the potential benefits of exercise for sleep [10]–[13] and its synchronising effects on the biological clock [14], [15].

The sleep-wake disturbances accompanying mood disorders can take heterogeneous forms including difficulties falling or staying asleep and/or frequent early morning awakenings, prolonged or reduced sleep time, non-restorative sleep, as well as abnormal timing and reduced amplitude of the sleep-wake cycle [16]–[23]. Some of these disturbances have been found to correlate with psychological distress and the intensity of depressive symptomatology, and to be risk factors for depression onset and recurrence [24]–[27]. Furthermore, specific interventions targeting daytime activity [28], [29] and/or sleep and circadian rhythms [30], [31] have been found to improve mood in persons with depressive syndromes, suggesting that components of the sleep-wake cycle may be modifiable pathogenetic factors for mood disorders. While these types of interventions are gaining increasing attention [32]–[35], there is a need to refine our understanding of the various sleep and circadian profiles associated with depression in order to more appropriately tailor novel sleep and circadian based interventions.

Some of the heterogeneity in the sleep-wake profiles among persons with mood disorders could be associated with age-related changes. Sleep and circadian rhythms change dramatically throughout the normal ageing process. In children and adolescents, total sleep time progressively decreases with age, while sleep consolidation indexes remain fairly stable [36], [37]. The onset of puberty is accompanied by a delay in the sleep-wake cycle [38], while the transition from adulthood to middle and old age is characterised by a shift to earlier sleep-wake schedules, poor sleep consolidation, reduced total sleep time, disorganised circadian rhythms and decreased circadian amplitude [37], [39]–[44]. These changes are likely to operate through age-related modifications in hormonal regulation, brain structure and function, sleep homeostasis as well as the timing and intensity of circadian signals [45]–[49], all factors that are also often affected by mood disorders.

The reduced daytime activity levels and the marked sleep and circadian disruptions linked to mood disorders could possibly interact with these age-related changes, thereby contributing to different sleep-wake profiles. For instance, earlier age of depression onset has been linked with later chronotypes (i.e. eveningness preference) in persons with bipolar disorder [50], later age of onset has been linked with poorer sleep consolidation in late-life depression [51] and reduced sleep consolidation during perimenopause has been linked with the emergence of mood disturbances [52]. Studies focussing on specific age groups revealed that young persons with mood disorders have delayed sleep-wake cycles, but that disruptions to sleep consolidation and total sleep time are not pronounced [53]–[55]. Conversely, those with late-life depression show poorer sleep consolidation compared to age-matched controls, even when depressive symptoms have largely remitted [51]. Some polysomnographic studies have compared samples of depressed patients and healthy controls ranging from youth to older age and found that the sleep of depressed patients shows signs of accelerated ageing with more pronounced sleep alterations [56]–[58]. However these studies were limited to a single night of sleep recording and did not consider possible changes in habitual sleep-wake schedule and patterns. To our knowledge, no study has directly compared objective measures of the sleep and activity cycles over multiple days in persons with mood disorders across various ages.

The current study used actigraphy to assess the influence of age and depressive symptoms severity on 24-hour activity profiles and sleep-wake patterns in patients with a history of mood disorders ranging from 12 to 90 years old. It was hypothesised that the profile of 24-hour activity and sleep-wake disturbances would differ across the various age groups. More specifically, depressive symptoms severity was expected to be associated with more pronounced phase delay during youth, as well as reduced sleep duration and consolidation and disorganisation of circadian rhythms in older age.

## Materials and Methods

### Ethics Statement

All participants were provided with a participant’s information statement document before meeting with someone from the research team who explained the study and answered any questions. This document stated that participation in this research would be entirely voluntary and that their decision whether to take part or not to take part (or to take part and then withdraw) in this study, would not affect their treatment or relationship with professionals at the Brain & Mind Research Institute. All participants gave their written informed consent prior to taking part in this study. Written consent was obtained from the parents or legal guardians of all participants who were younger than 16 years old. The study protocol was approved by the Human Research Ethics Committee of the University of Sydney, and was conducted according to the declaration of Helsinki.

### Participants

Two hundred and thirty-eight help-seeking persons with a lifetime history of mood disorders were selected from three pools of patients recruited from specialised assessment and early intervention services (Youth Mental Health Clinic [59], [60] and Healthy Brain Ageing Clinic at the Brain & Mind Research Institute, Camperdown, Sydney, Australia, and *headspace*, Campbelltown, Sydney, Australia). Sample characteristics are presented in Table 1. These participants were aged between 12 and 90 years and divided into five age groups: 12–19 y.o., 20–39 y.o., 40–59 y.o. and ≥60 y.o.

**Table 1:** Demographic and depression characteristics stratified by age.

	Age Group			
	12–19 y.o.	20–39 y.o.	40–59 y.o.	≥60 y.o.
n	47	90	49	52
Gender (%females)	78.7	58.9	63.3	55.8
Age (Mean (SD))	16.5 (2.0)	25.4 (5.1)	50.8 (5.1)	68.9 (7.0)
HDRS (Mean (SD)	12.8 (7.0)	14.1 (6.9)	9.4 (7.6)	7.3 (5.7)

HDRS: Hamilton Depression Rating Scale total score, SD: standard variation.

Lifetime mood disorder diagnoses (major depressive disorder or bipolar disorder) were established by a research psychologist or psychiatrist using DSM-IV-R criteria. Depression severity was rated using the Hamilton Depression Rating Scale [HDRS, 17-items; 61]. For some of the analyses, participants were divided in two groups according to depressive symptoms severity: symptomatic (HDRS≥8, n = 127) or asymptomatic (HDRS<8; n = 65). HDRS data was missing for 46 participants (4 from the 12–19 y.o. group, 20 from the 20–39 y.o. group, 19 from the 40–59 y.o. group and 3 from the ≥60 y.o. group). Exclusion criteria were: history of stroke; neurological disorder; head injury with loss of consciousness ≥30-minutes; medical condition known to affect cognition (e.g. cancer, dementia) and other psychiatric illness.

### Procedures

Actigraphy is an ambulatory monitoring technique that can be used to track daytime activity and nighttime sleep over multiple days. Motion changes detected every 30 or 60 seconds by a multidirectional accelerometer provide an objective measure of the intensity and timing of physical activity across the 24 hours cycle. Furthermore, based on established criteria to define sleep and wake states, actigraphy can be used to estimate the timing, length and consolidation of habitual sleep periods.

Actigraphy (Actiwatch-64/L/2/Spectrum, Philips Respironics, USA) was conducted for a period ranging between five and 22 days. Participants also completed a sleep diary during this period. Data from Actiwatch-L was collected over 1-minute epochs and data from all other actimeter models was collected over 30-second epochs. Sleep-wake detection was conducted automatically with Actiware 5.0 software (Philips Respironics) using the medium sensitivity threshold. Adjustments were made when necessary by qualified technicians using visual inspection and sleep diaries. Sleep estimations based on actigraphy remain indirect because this technique does not directly measure the brain’s vigilance state, but for descriptive purposes, actigraphy measures are defined here as ‘sleep'.

The following actigraphic sleep variables were calculated: i) sleep onset/offset (timing of the onset/offset of the rest episode), ii) time in bed (total duration of the rest episode), iii) total sleep time (amount of time scored as “sleep” within the rest episode), iv) wake after sleep onset (WASO: amount of time scored as “sleep” within the rest episode), and v) sleep efficiency (‘total sleep time’/‘time in bed’)*100). Herein, sleep onset latency was not integrated in the time in bed period (and thus in sleep efficiency calculations) because sleep diaries and event markers were not available for all participants.

**Figure 1:**
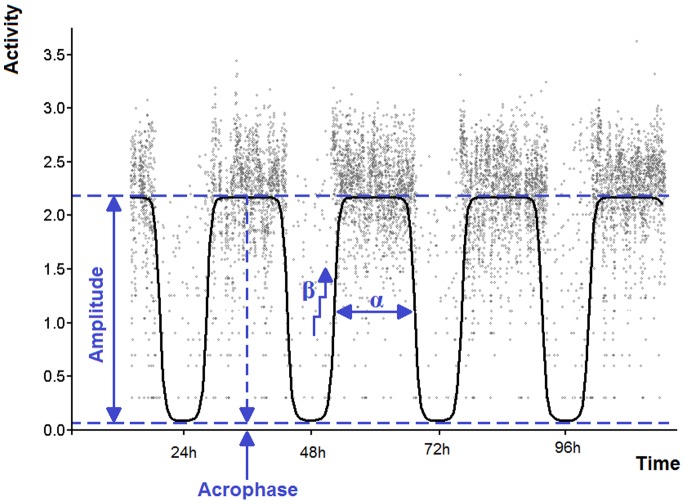
Example of activity cycle parameters derived from the extended Cosinor analysis. Each point represents a recording of activity intensity at a given time and the full line represents the fitted extended Cosinor curve. Nonlinear least-squares regression was used to fit actigraphy datasets to the model. The abscissa denotes time and the ordinate indicates activity intensity. Amplitude: difference between the peak and trough) of the fitted curve. Acrophase: time when the activity cycle reaches peak value. α: relative width of the curve at the middle of the peak. β: indicator of the steepness of the rise and fall of the curve. The coefficient of determination (or R^2^; not illustrated here), a measure reflecting the goodness of fit, was used as an indicator of circadian rhythmicity.

To characterise the circadian profile of the activity-rest cycle, individual actigraphy datasets were fitted to an extended Cosinor model [62] using nonlinear least-squares regression in Prism 4 (GraphPad Software, CA, USA). This allowed the estimation of five circadian curve parameters for each participant (Figure 1): i) amplitude (difference between the peak and trough of the fitted curve, herein estimating the range of activity levels across the 24 hr period)), ii) acrophase (a phase marker indicating the time when the fitted curve reaches its peak (i.e. time of maximal activity levels across the 24 h)), iii) α (relative width of the curve at the middle of the peak; negative values indicate that the active portion of the cycle is wider than its inactive portion), iv) β (indicator of the steepness of the rise and fall of the curve; higher values indicate steeper rise and fall) and v) circadian rhythmicity index (coefficient of determination (or R^2^), a goodness of fit measure for which higher values indicate smaller discrepancies between actigraphy data and values predicted by the Cosinor model; considered to be an indicator of the strength of circadian rhythmicity).

### Statistical Analyses

Kruskal-Wallis tests were conducted on HDRS scores to assess age and gender differences and t-tests were used to assess gender differences for actigraphy variables. Actigraphy variables were also submitted to two-way ANOVAs with two independent factors: depressive symptoms severity (i.e. symptomatic and asymptomatic) and age (12–19 y.o., 20–29 y.o., 30–39 y.o., 40–59 y.o. and ≥60 y.o.). Tukey's Honestly Significant Difference tests were used to decompose main effects. ANCOVAs controlling for gender were conducted on all variables for which significant effects were found. Univariate Pearson correlations were conducted between actigraphy variables, age and HDRS. Those actigraphy variables that were significantly associated with age and HDRS at the univariate level were then submitted to a standard multiple regression model (‘enter’ method). Semi-partial correlations were computed to assess the relative contributions of age and depression severity to actigraphy variables. Regression analyses were based on pairwise comparisons. All statistical analyses used an alpha of 0.05 and were conducted with Statistica 6.1 software (StatSoft Inc, USA).

## Results

### Sample Characteristics

Sample characteristics for all participants are reported in Table 1. A main age effect (H = 32.9, p≤.001) showed that the 12–19 y.o. group had significantly higher HDRS scores than the ≥60 y.o. group (p≤.001) and that the 20–39 y.o. group had significantly higher HDRS scores than the two older groups (all p≤.009). HDRS scores did not differ significantly between male and female participants (H = 1.7, p = .189). Male participants had significantly later sleep onset time (t = 2.9, p = .004), shorter total sleep time (t = −2.1, p = .038) and lower circadian rhythmicity (t = −2.4, p = .019) compared to female participants. Actigraphy data did not converge with the Cosinor model for 16 participants, suggesting abnormal circadian rhythm of the sleep-wake cycle in these individuals. Of these participants, two were from the 12–19 y.o. group, three from the 20–39 y.o. group, five from the 40–59 y.o. group and six from the ≥60 y.o. group. Seven of these were from the asymptomatic group and seven from the symptomatic group (two of these 16 participants had missing HDRS data).

### Sleep-wake Cycle Profiles across Age and Depressive Symptoms Severity Groups

**Figure 2:**
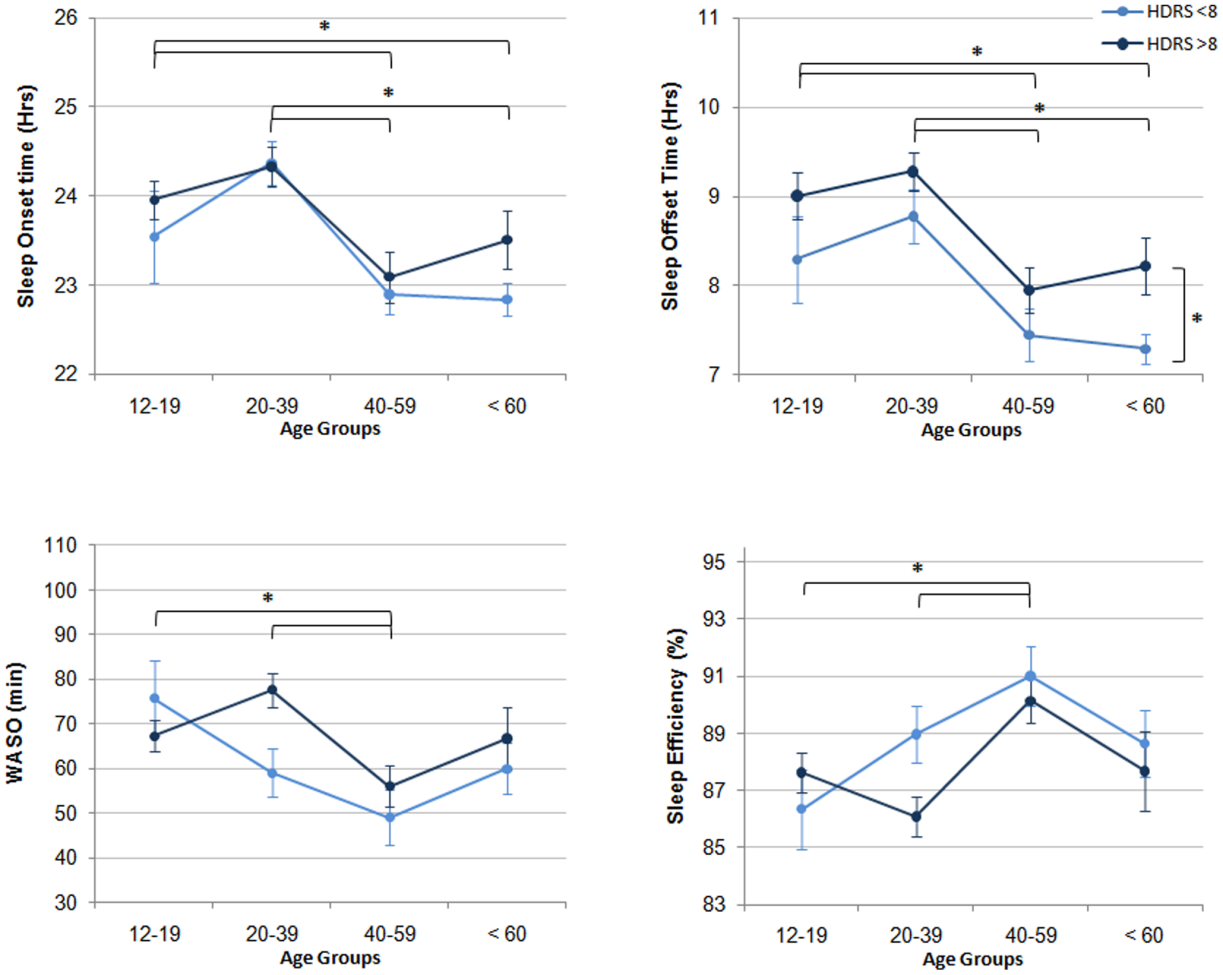
Sleep-wake variables across age groups and depressive symptoms severity levels. Horizontal bars indicate significant differences across specific age groups. Horizontal bars indicate significant depression severity effects. No significant interaction was found between age and depression severity. *p<.050.

**Figure 3:**
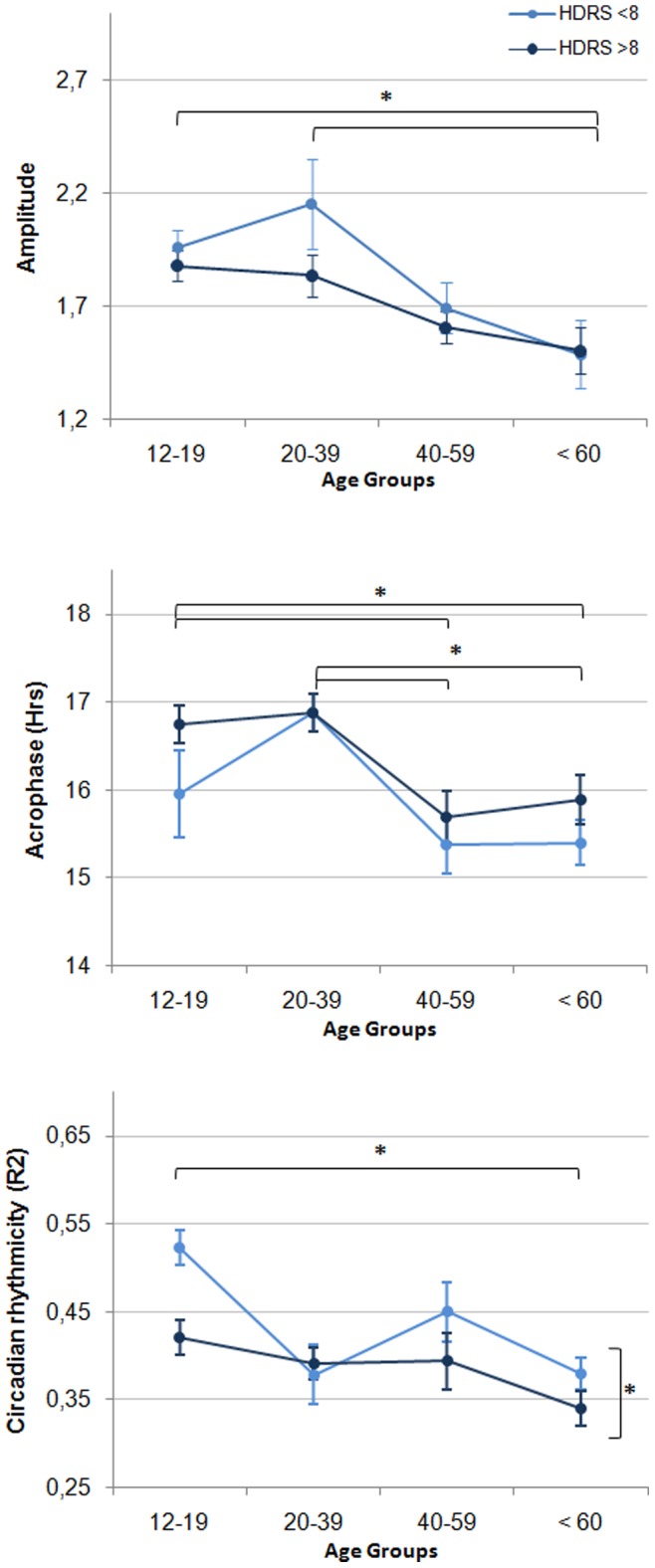
Circadian parameters of the activity-rest cycle derived from Cosinor analyses. Horizontal bars indicate significant differences across specific age groups. Horizontal bars indicate significant depression severity effects. No significant interaction was found between age and depression severity. *p<.050.

Figure 2 and 3 present significant age and depression effects revealed by the two-way ANOVAs. Main age effects were found for sleep onset and offset times, WASO, sleep efficiency, the amplitude, the acrophase, and the circadian rhythmicity index (Table 2, left section). In the 12–19 y.o. and 20–39 y.o. groups, sleep onset and offset times and the acrophase were found to be significantly delayed compared to that of the 40–59 y.o. and the ≥60 y.o. groups (all p≤.046). The 12–19 y.o. and 20–39 groups had significantly higher WASO and lower sleep efficiency compared to the 40–59 y.o. group (p<.045). The amplitude was significantly lower in the ≥60 y.o. group compared to the 12–19 y.o. and 20–39 y.o groups (p = .006 and p = .002 respectively). The circadian rhythmicity index was significantly lower in the ≥60 y.o. group compared to the 12–19 y.o. group (p = .006). Significant main effects of depressive symptoms severity were found for sleep offset time, time in bed, and the circadian rhythmicity index. Symptomatic participants had later sleep offset times (p<.004), spent longer time in bed (p<.036) and had lower circadian rhythmicity (p<.021) than asymptomatic participants. All these effects remained significant after controlling for gender. No significant interaction between age and depressive symptoms severity was found.

**Table 2:** Comparisons of actigraphy variables across age and depression severity groups and associations with depression severity and age.

	Mean (SD)								Two-way ANOVA (F)*n = 238*			Correlations (R)	
	12–19 y.o.		20–39 y.o.		40–59 y.o.		≥60 y.o.		HDRS	Age	HDRS *Age	HDRS*n = 192*	Age*n = 238*
	HDRS≥8	HDRS<8	HDRS≥8	HDRS<8	HDRS≥8	HDRS<8	HDRS≥8	HDRS<8					
Sleep_ON_	23.5 (1.7)	24.0 (1.2)	24.4 (.9)	24.3 (1.7)	22.9 (.8)	23.1 (1.2)	22.8 (1.)	23.5 (1.5)	1.8	7.7***	0.5	0.17*	−0.30***
Sleep_OFF_	8.3 (1.6)	9.0 (1.5)	8.8 (1.1)	9.3 (1.6)	7.4 (1.1)	7.9 (1.0)	7.3 (0.9)	8.2 (1.4)	8.3**	8.8***	0.2	0.28***	−0.39***
TiB (min)	525.1 (78.4)	543.0 (61.8)	504.3 (70.4)	537.0 (57.9)	513.3 (57.5)	531.5 (49.7)	507.2 (60.6)	523.0 (55.8)	4.5*	0.7	0.2	0.19**	−0.15*
TST (min)	449.3 (63.6)	472.5 (55.7)	445.3 (63.0)	459.4 (56.8)	464.2 (52.0)	475.5 (42.4)	452.7 (60.6)	456.3 (61.2)	1.9	0.6	0.2	0.09	−0.04
WASO (min)	75.7 (27.9)	67.3 (19.6)	59.0 (18.8)	77.7 (29.0)	49.1 (21.9)	56.0 (18.7)	60.0 (31.1)	66.7 (31.5)	1.9	3.1*	1.6	0.19**	−0.20**
SE (%)	85.9 (4.6)	87.1 (4.0)	88.5 (3.4)	85.6 (5.2)	90.5 (3.8)	89.6 (3.2)	88.1 (6.3)	87.2 (6.3)	1.1	3.1*	1.1	−0.16*	0.17**
Amplitude	1.96 (0.25)	1.88 (0.38)	2.15 (0.69)	1.84 (0.68)	1.69 (0.37)	1.61 (0.28)	1.48 (0.76)	1.50 (0.42)	1.4	6.3***	0.6	−0.05	−0.29***
Acrophase	15.5 (1. 6)	16.2 (1.2)	16.4 (0.7)	16.4 (1.6)	14.9 (1.1)	15.2 (1.1)	14.9 (1.3)	15.4 (1.1)	2.9	7.8***	0.5	0.19**	−0.32***
α	−0.34 (0.12)	−0.38 (0.17)	−0.48 (0.28)	−0.36 (0.29)	−0.33 (0.16)	−0.37 (0.14)	−0.36 (0.25)	−0.39 (0.14)	0.0	0.7	1.1	0.08	0.01
β	7.3 (2.8)	6.5 (4.6)	4.1 (2.1)	6.5 (3.6)	17.0 (32.6)	6.5 (2.8)	11.1 (19.7)	6.9 (3.8)	2.7	1.8	1.8	−0.10	0.15*
R^2^	0.52 (.06)	0.42 (.11)	0.38 (.12)	0.39 (.14)	0.45 (.11)	0.39 (.12)	0.38 (.09)	0.34 (.08)	5.4*	6.2**	1.5	−0.06	−0.13

Means, standard deviations (SD) and statistics for actigraphy variables across age groups and depression severity levels. HDRS: Hamilton Depression Rating Scale, Sleep_ON_: sleep onset, Sleep_OFF_: sleep offset, TiB: time in bed, TST: total sleep time, WASO: wake after sleep onset, SE: sleep efficiency, R^2^: circadian rhythmicity index.

*p<.050,

**p<.010,

***p<.001.

### Associations between the Sleep-wake Cycle, Depressive Symptoms Severity and Age

The right section of Table 2 presents correlations between actigraphy variables, HDRS score and age across all participants. Younger age and higher HDRS score were both found to correlate significantly with later sleep onset and offset times, longer time in bed, higher WASO, lower sleep efficiency and later acrophase (p≤.023). Lower circadian amplitude and higher β correlated significantly with older age, but not with symptoms severity (p≤.026).

### Predictive Factors for Disturbances of the Sleep-wake Cycle

Multiple regression analyses were conducted on sleep onset and offset times, time in bed, WASO, sleep efficiency and the acrophase (Table 3). Age was the strongest predictor for all sleep and activity phase markers, uniquely accounting for 6.8%, 9.6% and 7.0% of the total variance in sleep onset time, sleep offset time and the acrophase respectively (all p<.001). Age was also a significant predictor for WASO, accounting for 2.1% of its total variance (p = .043). HDRS score was a significant predictor of sleep offset time and time in bed, uniquely explaining 2.2% (p = .026) and 2.0% (p = .047) of their total variance respectively.

**Table 3:** Multiple regression model showing the contributions of age and depressive symptoms severity to sleep-wake cycle disturbances.

	Sleep_ON_	Sleep_OFF_	TiB	WASO	SE	Acrophase
*Predictors (β)*						
HDRS	0.07	0.16*	0.15*	0.13	−0.12	0.09
Age	−0.28***	−0.33***	−0.09	−0.15*	0.12	−0.28***
*Full model statistics*						
R	0.31	0.42	0.21	0.24	0.20	0.33
Adj R^2^	0.09	0.16	0.03	0.05	0.03	0.10
F	10.0	19.9	4.2	5.6	4.0	10.5
p	<0.001	<0.001	0.016	0.004	0.020	<0.001

Beta (β) values from multiple regression analyses and full model statistics. HDRS: Hamilton Depression Rating Scale, SleepON: sleep onset, SleepOFF: sleep offset, TiB: time in bed, WASO: wake after sleep onset, SE: sleep efficiency. Predictor significance:

*p<.050,

**p<.010,

***p<.001.

## Discussion

To our knowledge, this study is the first to examine the sleep-wake cycle and 24-hour activity profiles across various ages in persons with a history of mood disorders. It included patients with current depressive symptoms severity ranging from asymptomatic (i.e. remitted) to very severe, but all were seeking treatment for their mental health problems from specialist early intervention clinics. Partially supporting our hypothesis, our results indicate that the effects of age add to those of depression severity on the timing and rhythmicity of the sleep-wake cycle, but not on sleep duration. Younger age and higher symptoms severity were both associated with delayed sleep-wake schedule and daily activity peak, longer time in bed and poorer sleep consolidation. Notably, age was found to be a stronger predictor for sleep and activity phase markers than symptom severity.

Of significance, our data show that the timing of sleep onset/offset in persons with a lifelong history of mood disorders follows the general direction of normal age-related changes, with a delayed profile during youth and a subsequent phase advance from middle age to old age. Importantly, across all age groups, symptomatic participants were found to have later sleep offset times than asymptomatic participants. Consequently, the sleep-wake profile of young persons was characterised by the additive effects of the normal youth related sleep phase delay and the phase delay associated with their depression levels. This delay in the sleep-wake cycle was accompanied by a later peak of the 24-hour activity cycle in young compared to older participants. On the one hand, physical activity conducted late during the day can lead to difficulties in sleep initiation [13] and is recognised as a chronobiotic agent that can phase shift the sleep-wake cycle. On the other hand, individuals with a sleep phase delay may be more likely to shift their physical activity later to conform to their biological day. Consequently, the co-occurrence of a delayed sleep-wake cycle and a later peak of physical activity in younger participants with mood disorders could result from a bidirectional relationship between daytime activity and nighttime sleep.

This delayed profile is in line with the fact that young persons with mood disorders have a later sleep-wake cycle than healthy controls from the same age group [55] and the preference for later sleep and daily activity schedules commonly found in depressed persons, especially those at younger age [63]. These abnormalities in the timing of the sleep and activity cycles could be linked to delayed signals sent from the circadian clock. For instance, we recently observed delayed and reduced evening melatonin secretion in young persons with mood disorders [64]. Furthermore, in young persons with emerging mental disorders, we found that lower evening melatonin levels were associated with lower nighttime sleepiness [65]. Therefore, in the evening, the physiological state of young depressed individuals may be less conducive to appropriately timed sleep initiation, which would be likely to contribute to further sleep phase delay.

While both younger age and greater depression severity were associated with later sleep onset/offset times and later timing of the activity cycle peak, age was the strongest predictor of this phase delay. Interestingly, sleep offset was the only variable to be significantly predicted by both age and depression severity. Therefore, this specific phase marker may be especially sensitive to the additive effects of age and depression.

The amplitude of a circadian rhythm is thought to be strongly regulated by the strength of the signal sent by the biological clock. Herein, older participants (≥60 y.o.) had a lower range of activity intensities across the 24-hour cycle and disorganised rhythmicity of the 24-hour activity cycle. This is consistent with previous reports of a weakening of circadian output signals during old age, typically leading to lower circadian amplitude, lower entrainment stability and fragmented rhythms with altered temporal and structural order [66]. It is however important to mention that 24-hour variations in the activity cycle are influenced by several factors aside from the endogenous circadian drive. For instance, avolition and lack of energy are likely to contribute to the lower activity levels commonly found in persons with depression, which would in turn influence the amplitude of the 24-hour activity cycle. Nevertheless, our finding that symptomatic participants had more disorganised rhythm of the 24-hour activity cycle than those with milder levels of depressive symptoms suggests that some of these age-related effects may be further exacerbated by depression. Importantly, age-related disruptions of the biological clock are thought to have adverse consequences on general health which could potentially induce metabolic and cardiovascular dysfunctions as well as increased daytime sleepiness and fragmented nighttime sleep [67]–[69]. Similar adverse outcomes could be expected if the abnormalities found in the 24-hour activity profile of older persons with a history of mood disorders are partially reflective of endogenous circadian abnormalities or contribute to these endogenous circadian abnormalities. Of note, neither age nor depression severity were significantly correlated with the circadian rhythmicity index, suggesting that this effect may be less progressive than the additional effects of age and depression on the timing of the sleep-wake cycle.

Our sample of participants did not show the age-related reduction in total sleep time and sleep consolidation typically found in healthy samples, suggesting that the effects of depression on sleep quantity and quality (or other factors not explored in the current study such as use of antidepressant medications) may mask those of normal ageing.

Higher depression severity was found to be associated with higher WASO and lower sleep efficiency. This progressive decrease in sleep consolidation with higher symptoms severity is consistent with previous findings [70], [71]. However, our results do not support that of Lauer et al. [58] who found that sleep efficiency and WASO were more affected across ageing in persons with depression compared to healthy controls. Herein, no frank difference in WASO or sleep efficiency was found between symptomatic and asymptomatic participants. This may be due to a somewhat gradual decrease in sleep consolidation with increasing depression severity and/or to residual effects of depression persisting after remission. This notion is aligned with our prior data in older cognitively impaired cohorts showing residual effects of depression on sleep consolidation [72]. Similarly, other studies have found reduced sleep consolidation after successful psychotherapy for depression [18], [73]. However, considering the habitual worsening of sleep consolidation during older age, it is also possible that the higher proportion of older individuals in the asymptomatic group could have artificially reduced sleep consolidation indexes in this group, thereby attenuating the difference between the depressive symptoms severity groups. This discrepancy in depression severity across age groups could also explain why, as opposed to what is classically found in normal ageing, poorer sleep consolidation was found to be associated with younger age in our sample of participants.

Increasing efforts are being made to identify sleep and circadian variables that can act as specific biomarkers, predict illness trajectories and inspire novel sleep and circadian based treatments for mood disorders. As previously emphasised by others [56], the present findings reinforce the need to take age into account while investigating sleep in the context of mood disorders. Several descriptive studies which sought to establish the nature of sleep and circadian disturbances were based on groups of patients from large age ranges and may thus have confounded the impact of age. Most importantly, considering age may be a simple way to optimise the profiling and therapeutic value of sleep and circadian approaches. While polysomnographic markers of depression are well established in the adult and middle age population, most of these markers are less consistent in younger persons [58]. By providing information about habitual daily sleep-wake and activity patterns measured objectively across multiple days, actigraphy uncovered specific disturbances in the timing and rhythmicity of the sleep and activity cycles which could not have been detected by a single night of polysomnography. Herein, this allowed for the detection of biomarkers especially relevant for mood disorders in young and elderly persons.

This study is limited by the combination of various subtypes of mood disorders across the sample, the lack of a control group and the unequal age distribution across depression severity levels which is likely to reflect a selection bias in this specific sample. Nevertheless, some of the statistical tests controlled for this unequal distribution. Furthermore, while multiple regression models suggest that some variables may ‘predict’ or ‘contribute’ to variations in other variables, we note that the cross-sectional nature of the data collected in the current study does not allow to determine causality.

## Conclusion

Age modulates the profile of disturbances affecting the sleep and activity cycles in persons with a history of mood disorders. Depressive symptoms severity was associated with delayed sleep-wake and activity rhythms beyond the normal phase delay characteristic of youth and with further disorganisation of circadian rhythms in older age. Conversely, decreases in sleep consolidation may be a more stable effect of mood disorders across ageing. These findings highlight the importance of taking into account age as a modulator of sleep wake disturbances in those with mood disorders, and the need to recognise this when embarking on tailored sleep and circadian based therapies. Based on the current findings, one could hypothesise that mood disorders may be more responsive to phase shifting interventions in young persons and to interventions focussing on strengthening the circadian signal in older adults.
